# Structural Validity and Measurement Invariance of the Scale of Oral Health Outcomes for 5‐Year‐Old Children

**DOI:** 10.1111/ipd.70060

**Published:** 2025-11-29

**Authors:** Matheus França Perazzo, Ana Flávia Granville‐Garcia, Bianka Fernandes Delmônico, Andrea Sherriff, Roger Keller Celeste, Saul Martins Paiva, Georgios Tsakos

**Affiliations:** ^1^ Department of Oral Health Federal University of Goiás (UFG) Goiânia Brazil; ^2^ Postgraduate Program in Dentistry State University of Paraíba (UEPB) Campina Grande Brazil; ^3^ Glasgow Dental Hospital and School Glasgow UK; ^4^ Department of Preventive and Social Dentistry Federal University of Rio Grande Do Sul (UFRGS) Porto Alegre Brazil; ^5^ Department of Paediatric Dentistry Federal University of Minas Gerais (UFMG) Belo Horizonte Brazil; ^6^ Department of Epidemiology and Public Health UCL London UK

**Keywords:** cross‐cultural comparison, psychometrics, quality of life, statistical factor analysis, validation

## Abstract

**Background:**

Important psychometric approaches (structural validity, measurement invariance) remain underdeveloped in measuring oral health‐related quality of life, particularly for preschool children across diverse contexts.

**Aim:**

This study aimed to evaluate the structural validity of the child's self‐reported version of the Scale of Oral Health Outcomes for 5‐year‐old children (SOHO‐5) and test the measurement invariance from cultural and clinical/non‐clinical comparison perspectives.

**Design:**

Three datasets were analysed: two from Brazil and one from the United Kingdom (UK). One Brazilian dataset was derived from clinical data collection (*n*_br‐cl._ = 193), while the others were from non‐clinical epidemiological school‐based studies (*n*_br‐ncl._ = 768, *n*_uk‐ncl._ = 296). Dimensionality was tested through parallel analysis and confirmed by unidimensional indexes. Measurement invariance across datasets was tested via multi‐group Confirmatory Factor Analysis (CFA).

**Results:**

Unidimensionality was empirically confirmed for all three datasets. The multi‐group CFA tests reached partial scalar invariance threshold between the Brazilian and UK non‐clinical datasets. However, there was no scalar equivalence when comparing non‐clinical with clinical datasets, neither within Brazil nor between countries.

**Conclusion:**

The child's self‐reported version of the SOHO‐5 is a unidimensional oral health‐related quality‐of‐life measure that is psychometrically comparable across different cultures (partial scalar invariance), but not between clinical and non‐clinical groups.

## SUMMARY

- Why this paper is important to pediatric dentists
  ○ The child version of SOHO‐5 is a unidimensional oral health‐related quality‐of‐life measure that demonstrated robust evidence of structural validity to be used in paediatric populations.
  ○ Measurement invariance remains underexplored in the oral health‐related quality of life (OHRQoL) field, and this study advances the discussion for SOHO‐5 by employing robust psychometric methods to evaluate invariance across cultural and clinical/non‐clinical contexts. The results show that SOHO‐5 is psychometrically equivalent between different cultures, but not between clinical and non‐clinical groups.
  ○ Caution is needed when embarking on SOHO‐5 comparisons between clinical and non‐clinical populations in multicentre studies and meta‐analyses, even from the same cultural context.

## 1 Introduction

Oral health‐related quality of life (OHRQoL) is a construct concerning the subjective evaluation of oral health, functional and emotional well‐being, and expectations and satisfaction with care [1]. It is a complex construct influenced by the social, cultural and political context [2]. Since cognitive and social relationships represent an important stage of development during early childhood, oral health issues may have a negative impact on the OHRQoL of preschool children with far‐reaching detrimental consequences in the short‐ and long‐term [3, 4, 5]. However, OHRQoL scales for young children, particularly those that are self‐reported, were developed recently and chronologically later than the respective scales for older children, while their psychometric evaluation is still incomplete [6]. The reasons for this later and incomplete development and evaluation relate to conceptual challenges and the belief (of researchers) about the younger children's inability to report abstract domains [6, 7].

The child version of the Scale of Oral Health Outcomes for 5‐year‐old children (SOHO‐5) was the first scale to facilitate assessment of the OHRQoL of young children through self‐reports [5, 8]. Evidence supports that preschool children can report concrete domains of their quality of life, such as issues concerning dysfunction [9, 10]. Moreover, parents/caregivers may have incomplete knowledge to proxy‐report their children's health accurately due to their work routine, social life or the time the child stays at day‐care facilities [11]. Despite the theoretical and practical relevance of SOHO‐5 and its applicability even in national surveys [12], structural validity evidence is lacking. This refers to the empirical evaluation of the model dimensionality through, for example, Exploratory or Confirmatory Factor Analysis, which is absent for both the original version and most of its cross‐cultural adaptations [13]. Studies have tested the model fit of SOHO‐5 on samples of 69 (Dominican Republic version) [14], 173 (Myanmar version) [15] and 306 (Thailand) [16] children, through Confirmatory Factor Analysis based on an empirical expectation of unidimensionality. Therefore, advancing the comprehension of the structural validity is the next step in the consolidation of the SOHO‐5 among the OHRQoL scales for children.

Furthermore, the growth in cross‐cultural adaptations has brought forward a discussion about psychometric properties and requirements in different research fields, though still less so for OHRQoL [6]. The main question is to determine the extent to which those adaptations measure the same construct as the original version. This can be addressed by testing measurement invariance among the datasets from the different populations. Without it, there is no assurance that the scale truly assesses the same construct, even after following the translation, back‐translation and other cross‐cultural phases appropriately. Consequently, cross‐cultural comparisons can be misleading as the scale may tap on slightly different constructs in the different populations [17]. Besides, there is no evidence about how certain sampling or methodological characteristics of the target child population (e.g., being non‐clinical or clinical) may reflect on the measurement of the OHRQoL construct across cross‐cultural adaptations and/or different settings. Those knowledge gaps are not unique to SOHO‐5, but common to OHRQoL scales [6]. Employing appropriate psychometric approaches to acquire high‐quality evidence on validity may contribute to the best practice and effective use of a scale. Therefore, this study aimed to: (1) evaluate the structural validity of the child's self‐reported version of the SOHO‐5; (2) test the measurement invariance in terms of cultural and clinical/non‐clinical comparisons.

## 2 Methods

### 2.1 Datasets

Three cross‐sectional datasets were used in this study, two from Brazil and one from the United Kingdom (UK). All three samples had been collected in earlier independent studies conducted by different research groups and included authors of the present paper [5, 8, 18]. In addition to the cultural, sociodemographic and economic differences among the respective populations, there were also the following distinct clinical and epidemiological characteristics:

#### 2.1.1 Brazilian Non‐Clinical Dataset

A representative school‐based study was conducted in a sample of 768 children (47.5% female) enrolled in private and public preschools in Campina Grande, a city in northeastern Brazil. This city has a Human Development Index (HDI) of 0.72, classifying it as a municipality with high human development, but also presents a Gini coefficient of 0.586, reflecting significant economic inequality [19]. The prevalence of dental decay was 58.8% and 23.8% had previous experience of dental pain [18].

#### 2.1.2 Brazilian Clinical Dataset

Data were collected from a convenience sample of 193 children (45.1% female) who had not received dental treatment in the last 3 months, recruited from the University of São Paulo (USP) paediatric dental screening program in southeast Brazil. This city has a HDI of 0.81, indicating a higher human development than Campina Grande, but has a Gini coefficient of 0.645, which also reflects substantial economic inequality [18]. The prevalence of dental decay was 54.4%, and the previous experience of dental pain represented 59.1% of the sample [8].

#### 2.1.3 UK'S Non‐Clinical Dataset

This is a school‐based study with a convenience sample of 296 5‐year‐old children (55.0% female) derived from public schools included in the National Dental Inspection Programme in Glasgow, Scotland. Scotland have an HDI of 0.940 and a GINI of 0.326 [20]. One quarter of the sample had at least one dental decay, and 16.9% had some experience of dental pain [5].

The eligibility criteria for those studies comprised children with no systemic diseases (according to their parents'/caregivers' reports). Parents/caregivers of all participants provided written consent.

### 2.2 Measure

**FIGURE 1:**
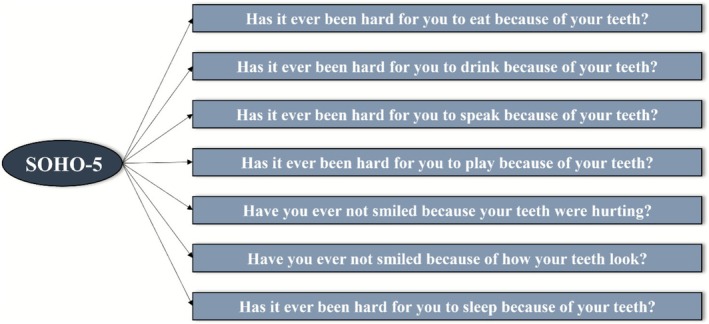
Conceptual unidimensional model of SOHO‐5 [5].

The child version of SOHO‐5 is a seven‐item, self‐reported OHRQoL scale. The questions refer to whether the condition or appearance of the teeth has affected key daily life functions [eating, drinking, speaking, playing, smiling (due to pain/due to appearance), sleeping] in 5‐year‐old children, with a three‐point response option (no = 0, a little = 1, a lot = 2). The final score ranges from 0 to 14, with higher scores denoting a poorer OHRQoL. The SOHO‐5 was conceptually developed as a unidimensional scale (Figure 1) [5].

### 2.3 Data Analysis Plan

Statistical analyses were performed in SPSS 22.0 (IBM Corp., Armonk, NY, USA), Mplus v. 8.8 and Factor 11.0.

#### 2.3.1 Structural Validity

Considering the lack of previous evidence of specific parameters from Exploratory Factor Analysis (EFA) for the SOHO‐5, Parallel Analysis was initially used to assess the dimensionality for the three datasets. The quality and effectiveness of factor score estimates were evaluated through Factor Determinacy Index (FDI), expected a posteriori (EAP) marginal reliability, and expected percentage of true differences (EPTD). When the FDI value is near one (> 0.90), the factor score estimates are good proxies for representing the latent factor scores. Similarly, an acceptable EAP marginal reliability (> 0.80) indicates precise measures of the reliability of the factor score estimates. The EPTD is recommended above 90%, and it estimates the percentage of differences between the observed factor score estimates that are in the same direction as the corresponding true differences. The unidimensionality of the construct (or closeness to it) was tested according to the following indices, and cut‐off values: Unidimensional Congruence (UNICO) > 0.95, Explained Common Variance (ECV) > 0.80 and Mean of Item Residual Absolute Loadings (MIREAL) < 0.30 [21]. The McDonald's omega (ω) coefficient measured the internal consistency of the scale under an acceptable lower bound limit of 0.70 [22].

As a preliminary step, measurement invariance requires the specification of the models. Therefore, the Confirmatory Factor Analysis (CFA) was employed through Diagonally weighted least squares (WLSMV) estimation. Model goodness of fit was assessed by the normed chi‐squared (χ^2^/df), Comparative Fit Index (CFI), Root Mean Square Error of Approximation (RMSEA) and the Standardised Root Mean Square Residual (SRMR). The following thresholds were adopted to adjudge model fit: χ^2^/df < 5.0, CFI > 0.90, RMSEA < 0.06 and SRMR < 0.10 for adequate fit; χ^2^/df < 2.0, CFI > 0.95, RMSEA < 0.06 and SRMR < 0.08 for acceptable fit [23].

#### 2.3.2 Measurement Invariance

Measurement invariance across datasets was tested via multi‐group CFA, contrasting the unidimensional first‐order constructs in three steps: Configural, metric (weak) and scalar (strong) invariance [24]. Configural invariance represents the baseline, which assumes that groups hold the same conceptual framework without equality constraints on any parameter. Metric invariance requires equivalence among the factor loadings, meaning that each item contributes to the latent construct to a similar degree across the three groups. Scalar invariance is tested by constraining intercepts to be equal among the groups. This level of invariance is necessary for comparing latent mean differences across groups, as it pertains to the underlying construct rather than the raw mean scores [24]. If scalar invariance is achieved, scores would have the same unit of measurement among the datasets [25, 26].

Both metric and scalar invariance were tested by comparing the corresponding nested models. Changes within −0.010 units for the CFI and 0.015 units for the RMSEA support the invariance of the more restricted (nested) model relative to the less restrictive one. Similarly, changes of SRMR within 0.030 units are indicative of metric invariance, while changes within 0.010 units are indicative of scalar invariance [25]. Despite the fit indices' importance in evaluating the invariance, there is no consensus on the best indices or cut‐off values [27].

## 3 Results

The parallel analysis indicated the unidimensionality of the construct measured through SOHO‐5, which was corroborated by the UNICO > 0.95, ECV > 0.80 and MIREAL < 0.30 across the three datasets. Besides, the quality and effectiveness of factor score estimates (FDI, EAP marginal reliability and EPTD) were considered adequate. The internal consistency reached satisfactory values for the *ω* (0.875–0.936) coefficients (Table 1).

**TABLE 1:** Summary of the unidimensional model characteristics of Brazilian (Non‐clinical and Clinical) and UK datasets.

		SOHO‐5 CHILD VERSION		
		Brazil (ncl.)	Brazil (cl.)	UK (ncl.)
Exploratory	FDI	0.972	0.960	0.954
	EAP marginal reliability	0.944	0.922	0.910
	EPTD	94.9%	93.6%	93.0%
Dimensionality	UniCo	0.972 (0.938–0.991)	0.982 (0.973–0.993)	0.963 (0.942–0.986)
	ECV	0.907 (0.870–0.944)	0.874 (0.845–0.912)	0.847 (0.815–0.904)
	MIREAL	0.178 (0.145–0.218)	0.253 (0.181–0.310)	0.275 (0.214–0.331)
Reliability	McDonald's Omega	0.936	0.904	0.875

Abbreviations: cl., clinical dataset; EAP, expected a posteriori; ECV, Explained Common Variance; EPTD, Expected Percentage of True Differences; FDI, Factor Determinacy Index; MIREAL, Mean of Item Residual Absolute Loadings; ncl., non‐clinical dataset (school‐based); UniCo, Unidimensional Congruence.

The unidimensional models tested through CFA yielded an equally acceptable fit to the data. The residual variance was inspected, and no covariance was necessary to be identified in the model. However, the RMSEA had a confidence interval broader than the cut‐off point recommended in the Brazilian (clinical) and UK datasets. Factor loadings were higher than 0.60 for all indicators except for the item ‘Hard to eat’ in the UK dataset (Table 2).

**TABLE 2:** Confirmatory factor analysis Brazilian (non‐clinical and clinical) and UK datasets.

	Brazil (ncl.)	Brazil (cl.)	UK (ncl.)
	λ (95% CI)	λ (95% CI)	λ (95% CI)
Hard to eat	0.742 (0.689–0.796)	0.655 (0.536–0.774)	0.482 (0.335–0.630)
Hard to drink	0.850 (0.806–0.893)	0.725 (0.568–0.882)	0.727 (0.599–0.855)
Hard to speak	0.879 (0.840–0.917)	0.805 (0.653–0.958)	0.674 (0.511–0.836)
Hard to play	0.869 (0.831–0.906)	0.782 (0.665–0.900)	0.894 (0.821–0.968)
Ever not smiled (pain)	0.858 (0.820–0.897)	0.886 (0.807–0.965)	0.750 (0.631–0.868)
Ever not smiled (appearance)	0.687 (0.615–0.759)	0.682 (0.548–0.815)	0.749 (0.625–0.872)
Hard to sleep	0.865 (0.826–0.904)	0.815 (0.709–0.920)	0.661 (0.533–0.789)
χ^2^/df	1.027	1.693	1.459
CFI	0.999	0.986	0.983
RMSEA (95% CI)	0.006 (0.000–0.036)	0.060 (0.003–0.100)	0.039 (0.000–0.074)
SRMR	0.015	0.054	0.059

Abbreviations: CFI, Comparative Fit Index; cl., clinical dataset; df, degrees of freedom; ncl., non‐clinical dataset (school‐based); RMSEA, Root Mean Square Error of Approximation; SE, standard error; SRMR, Standardised Root Mean Square Residual; λ, standardised loading; χ^2^, Chi Square.

Measurement invariance was tested through two‐way comparisons to explore the presence of one possible non‐invariance among the datasets, assessing differences across countries and also between non‐clinical and clinical samples (Table 3). Fit indices of the baseline model for the three‐way analysis were in line with the hypothesis of configural measurement invariance. However, in the Brazil vs. UK non‐clinical comparison, ΔRMSEA (0.018) slightly exceeded the commonly accepted cut‐off of 0.015. Given that all other indices were within recommended values, we further inspected modification indices, which suggested local misfit for the item ‘have you ever not smiled because of how your teeth look?’ (‘ever not smiled (appearance)’). Allowing this parameter to vary across groups led to a partial metric invariance model, with improved fit (ΔCFI = 0.003; ΔRMSEA = 0.005; ΔSRMR = 0.009). Following this adjustment, scalar invariance was tested by also freeing the thresholds of this item, resulting in partial scalar invariance (ΔCFI = −0,003, ΔRMSEA = 0.000, ΔSRMR = 0.001). There were no scalar equivalences between the non‐clinical and clinical datasets.

**TABLE 3:** Measurement invariance analyses between the Brazilian (non‐clinical and clinical) and UK datasets.

Models	χ^2^ (df)	CFI	ΔCFI	RMSEA (90% CI)	ΔRMSEA	SRMR	ΔSRMR
Brazil (ncl.) and UK (ncl.)							
Configural	36.080 (28)	0.999	—	0.023 (0.000–0.043)	—	0.034	—
Metric	63.867 (34)	0.995	−0.004	0.041 (0.025–0.056)	**0.018**	0.052	0.018
Metric (partial)^a^	46.682 (33)	0.998	0.003	0.028 (0.000–0.045)	0.005	0.043	0.009
Scalar (partial)^b^	88.596 (47)	0.995	−0.003	0.036 (0.021–0.050)	0.000	0.044	0.001
Brazil (ncl.) and Brazil (cl.)							
Configural	39.668 (28)	0.998	—	0.029 (0.000–0.049)	—	0.028	—
Metric	37.572 (34)	0.999	0.001	0.015 (0.000–0.037)	−0.014	0.032	0.004
Scalar	**134.864 (47)**	0.986	−0.013	**0.062** (0.050–**0.075**)	**0.047**	0.038	0.006
Brazil (cl.) and UK (ncl.)							
Configural	43.918 (28)	0.985	—	0.048 (0.016–**0.074**)	—	0.057	—
Metric	52.926 (34)	0.982	−0.003	0.048 (0.019–**0.072**)	0.000	0.070	0.013
Scalar	95.016 (47)	0.954	**−0.028**	**0.065** (0.046–**0.083**)	**0.017**	0.074	0.004

*Note:* Bold values indicate fit indices or changes in fit indices that exceeded recommended cut‐off criteria.

Abbreviations: CFI, Comparative Fit Index; cl., clinical dataset; df, degrees of freedom; ncl., non‐clinical dataset (school‐based); RMSEA, Root Mean Square Error of Approximation; SE, standard error; SRMR, Standardised Root Mean Square Residual; λ, standardised loading; χ^2^, Chi Square.

^a^ Partial metric invariance was adopted because the ΔRMSEA value (0.018) slightly exceeded the commonly accepted cut‐off of 0.015. The loading of item ‘ever not smiled (appearance)’ was allowed to vary across groups, as modification indices (MI) indicated localised misfit (MI≈5.9 for both loadings).

^b^ Scalar invariance was tested by additionally allowing the thresholds of item ‘ever not smiled (appearance)’ to vary across groups, consistent with its factor loading being released at the metric level.

## 4 Discussion

Using three different datasets from two countries, the results of this study support the theoretically assumed structure of the child version of SOHO‐5 as unidimensional. Although previous studies [14, 15, 16] reported an adequate unidimensional model fit, the small sample sizes or extensive use of error covariances may have influenced the accuracy of the model's fit. Besides, the present study found equivalence to non‐clinical datasets but non‐equivalence between non‐clinical and clinical datasets on the scalar level. Measurement invariance is in a nascent stage in the OHRQoL field, and very few studies have tested it between genders [28], time [29], and groups from the same continent through multi‐group CFA [30]. The present study advances the discussion not just for SOHO‐5 but also for the OHRQoL research in general, proposing an empirical evaluation of the perspective of dynamic constructs through testing cultural and clinical/non‐clinical invariances.

Evidence of structural validity and measurement invariance for SOHO‐5 was presented using state‐of‐the‐art methods [24]. Psychometric analysis to investigate abstract constructs in oral health has progressed in the last decades; however, psychometric properties are predominantly assessed through traditional approaches and this may lead to endorsing suboptimal scales. Poor structural validity evidence is a concern for many Patient‐Reported Outcome Measures (PROMs), with the main issues referring to a lack of assessment of latent traits, inadequate choices to test the model, and factor extraction methods that overestimate the number of dimensions [31]. Moreover, psychometric rigour also impacts the comparability of a construct evaluated in different contexts.

Although quality of life is generally conceptualised through grounded multidimensional models, the SOHO‐5, and many short OHRQoL scales, have been developed without documenting their dimensionality [6, 32]. For the SOHO‐5 in particular, unidimensionality was assumed but not tested. Finding fewer factors in the measurement model can derive from different reasons: (1) the construct established on the conceptual model is not distinguishable exactly as thought; (2) the items developed to represent the anticipated dimensions are not pure measures of these dimensions; (3) there are not enough items to bring out the aspect of a specific dimension [33]. The last reason may better justify the unidimensionality of the child's self‐reported SOHO‐5, but this is also partly due to the specific characteristics of the target population. The SOHO‐5 was planned to be short considering the cognitive ability of 5‐year‐olds [5]. This scale differs from others in which the time to answer is prioritised over the conceptual model [34, 35].

Fit indices indicated an adequate model fit for all datasets. The larger RMSEA's confidence interval for the UK and Brazilian (non‐clinical) datasets may be explained by the small degrees of freedom (df) and sample sizes as in such cases, the RMSEA cut‐off point too often falsely indicates a poorly fitting model [36]. Therefore, the limitations of RMSEA were considered in the interpretations of model fit and subsequent measurement invariance.

The non‐invariance between clinical and non‐clinical (school‐based) populations suggests that the construct comparison among those environments might be unreliable [24]. It is highly probable that there is a different perspective towards determining OHRQoL when a child is seen for dental treatment than when it reports on it in a school setting while not in a dental patient capacity. The non‐invariance was found not only when comparing the construct across a clinical and an epidemiological sample between the two countries but also when doing this comparison within those two different samples within a country (Brazil). These results are divergent from a previous study that evaluated the invariance of the OHIP‐14 scale in China between clinical and non‐clinical data [30], though the non‐clinical sample comprised university students, and clinical profiles were not reported for each group. Future studies should consider the possible non‐invariance of OHRQoL when interpreting results from clinical samples and non‐clinical (general) populations of children.

From a cross‐cultural perspective, the invariance between the Brazilian and UK datasets was clearly documented when considering both datasets from a similar environment (non‐clinical samples). However, this invariance was established at a *partial* level: the *item* ‘ever not smiled (appearance)’ did not demonstrate metric and scalar invariance across groups. Among the SOHO‐5 items, it is the only one capturing a purely aesthetic dimension, while all others address primarily functional (e.g., eating, speaking, playing) or pain‐related impacts. In school‐based populations, children's responses to appearance‐related items are likely influenced by cultural and social norms. Contextual differences plausibly explain the lack of invariance for this specific item [37]. Importantly, after accounting for this localised misfit, partial scalar invariance was achieved, enabling valid cross‐cultural comparisons while maintaining methodological transparency [24, 25].

It is also worth noting that the item ‘Hard to eat’ showed a slightly weaker loading (0.48) in the UK sample. Nonetheless, the loading was not flagged by any relevant modification indices and did not affect the invariance results. This may reflect a relatively lower prominence of functional eating difficulties in UK school‐based populations, rather than a threat to the structural validity of the scale.

The practice of testing measurement invariance in cross‐cultural adaptations is neither standard nor frequent [6]. However, documenting measurement invariance (partial or full) in a cross‐cultural adaptation guarantees that the scale truly assesses an equivalent construct of OHRQoL, thereby facilitating cross‐cultural comparisons [17]. Cross‐cultural adaptations should follow robust psychometric analysis, and this has the potential to reveal more relevant information than simply assuming cross‐cultural equivalence [38]. Studies on measurement invariance of established scales can corroborate important evidence to guide their use and interpretation.

This study has some limitations that should be considered when interpreting the results. Two datasets used in the analysis comprised a convenience sample. Although rigorous psychometric methods were employed, the relatively small sample sizes of the Brazilian non‐clinical dataset may have influenced the model fit indices, such as RMSEA, indicating the need for careful contextual interpretation. In addition, the Brazilian clinical dataset (*n* = 193) was substantially smaller than the combined non‐clinical samples, which may have reduced the stability of parameter estimates and contributed to the non‐invariance observed between clinical and non‐clinical groups. Further studies should generalise these findings to other cultures and populations with representative samples. Future studies should also continue to investigate for which groups/environments the relevant scales are meaningfully comparable, so that the best measurement practice can be advanced. The use of Item Response Theory can be an alternative approach to identify differential item functioning that compromised the comparability between clinical and non‐clinical.

Beyond the methodological contribution, these findings have direct implications for both research and clinical practice. The evidence of partial scalar invariance across Brazilian and UK non‐clinical datasets indicates that latent mean comparisons of OHRQoL can be meaningfully conducted between these countries, provided that appearance‐related items are interpreted with caution. For clinical settings, the lack of scalar invariance between clinical and non‐clinical groups suggests that scores should not be directly comparable across these contexts [25]. Children assessed in a clinical setting may be influenced by prior treatment experiences, treatment expectations, or the presence of oral disease, whereas children in school‐based contexts may frame their answers more strongly in relation to peer interactions. In practice, this means that interpreting group differences between school‐based and patient populations as solely reflecting differences in OHRQoL should be avoided; instead, part of these differences may be due to measurement non‐equivalence [24]. This insight underscores the importance of considering measurement invariance before comparing PROM scores across different populations and highlights the need for further research to identify items or dimensions that may be context‐specific. At the same time, these conclusions should be interpreted with caution, in line with the earlier discussion on the relatively small size of the Brazilian clinical sample.

Those results might indicate that quality‐of‐life research should be more parsimonious about the dynamic construct perspective. The measurement of health does not need many different new scales for each culture if invariance is proven between versions of an adequate and well‐established scale. Understanding the possibilities of comparability of PROMs is essentially about following an inclusive science path.

## Author Contributions

M.F.P., A.F.G.‐G., B.D., A.S., R.K.C., S.M.P. and G.T. conceptualised the manuscript and M.F.P., A.F.G.‐G., A.S., S.M.P. and G.T. provided the datasets. M.F.P. wrote a draft of the manuscript. All authors interpreted results and revised critically the manuscript. All authors approved final version.

## Funding

Funding was provided by the National Council for Scientific and Technological Development (CNPq)—No 10/2023, under grant No. 420299/2023‐8; Research Support Foundation (FUNAPE) from Goiás—No 01/2022 and by the Coordenação de Aperfeiçoamento de Pessoal de Nível Superior—Brazil (CAPES)–Finance Code 001.

## Ethics Statement

This study was conducted in accordance with the ethical principles outlined in the Declaration of Helsinki. Ethical approvals were obtained from the following committees: Human Research Ethics Committee of the State University of Paraiba (no 38937714.0.0000.5187); West Glasgow Ethics Committee (no 07/S0709/60) and USP Ethics Committee in Research (no 174/2009).

## Conflicts of Interest

The authors declare no conflicts of interest.

## Data Availability

Data available on request.
